# Importance of active case detection in a malaria elimination programme

**DOI:** 10.1186/1475-2875-13-186

**Published:** 2014-05-23

**Authors:** Renu Wickremasinghe, Sumadhya Deepika Fernando, Janani Thillekaratne, Panduka Mahendra Wijeyaratne, Ananda Rajitha Wickremasinghe

**Affiliations:** 1 Department of Parasitology, Faculty of Medical Sciences, University of Sri Jayewardenepura, Gangodawila Nugegoda, Sri Lanka; 2 Department of Parasitology, Faculty of Medicine, University of Colombo, P O Box 271, Kynsey road, Colombo 8, Sri Lanka; 3 Tropical and Environmental Disease and Health Associates, 3 Elibank Road, Colombo 5, Sri Lanka; 4 Department of Public Heath, Faculty of Medicine, University of Kelaniya, P O Box 6, Thalagolla Road, Ragama, Sri Lanka

**Keywords:** Malaria elimination, Active case detection, Mobile malaria clinics, Sri Lanka

## Abstract

**Background:**

With the aim of eliminating malaria from Sri Lanka by 2014, the Anti-Malaria Campaign of Sri Lanka (AMC) sought the support of Tropical and Environmental Disease and Health Associates Private Limited (TEDHA), a private sector organization. In 2009, TEDHA was assigned 43 government hospitals in the district of Mannar in the Northern Province and in districts of Trincomalee, Batticaloa and Ampara in the Eastern Province to carry out malaria surveillance to complement the surveillance activities of the AMC. Passive case detection (PCD), activated passive case detection (APCD) and active case detection (ACD) for malaria have been routinely carried out in Sri Lanka.

**Methods:**

The active case detection programme of TEDHA involves screening of populations irrespective of the presence of fever or any other signs or symptoms of malaria to detect infections and residual parasite carriers. ACD is done by TEDHA in a) high risk populations through mobile malaria clinics including armed forces personnel and b) pregnant females who visit antenatal clinics for asymptomatic malaria infections during the first trimester of pregnancy. Populations are selected in consultation with the Regional Malaria Officer of the AMC thus avoiding any overlap with the population screened by the government.

**Results:**

TEDHA screened 387,309 individuals in the four districts for malaria by ACD including high risk groups and pregnant women between January 2010 and December 2012. During this period seven individuals were diagnosed with *Plasmodium vivax* infections and one individual was detected with a mixed infection of *P. vivax* and *Plasmodium falciparum*. All eight cases were detected by ACD carried out by mobile malaria clinics among high risk groups in the Mannar district.

**Conclusion:**

The progress made by Sri Lanka in the malaria elimination drive is largely due to increased surveillance and judicious use of control methods which has resulted in zero indigenous malaria cases being reported since October 2012. ACD played a major role in interrupting malaria transmission in the country.

## Background

Over the past 150 years, the strategy to gradually eliminate malaria worldwide has shown remarkable progress [1]. In spite of 3 million lives being saved between 2000–2012, an estimated 627,000 people die from malaria each year [2]. Increased resources has resulted in a dramatic expansion and scaling up of malaria control interventions with subsequent reductions in disease burden in some parts of the world [1]. Malaria elimination is the ultimate goal of any malaria control programme and requires commitment at the highest level [3].

Strong malaria surveillance systems are fundamental to both programme design and implementation [2]. Asymptomatic parasite carriers provide a reservoir of infection in low-endemic countries that may contribute to continuous low-grade transmission of the disease and ignite devastating epidemics. With emphasis being given to track every malaria case in a surveillance system [4], scaling up diagnostic testing to ensure detection of asymptomatic cases and treating them so as to interrupt indigenous transmission, is a major challenge for the successful implementation of a malaria elimination programme in any malaria-endemic country.

Following the devastating malaria epidemic which occurred in Sri Lanka in 1934-35 [5], the country was successful in bringing down the number of cases to 17 in 1963. However, due to the early withdrawal of control measures such as indoor residual spraying as per the strategy adopted in the consolidation phase of the malaria eradication programme at that time, poor surveillance and withdrawal of funding for malaria control as malaria was not considered a priority due to the decreased disease burden, a major epidemic was recorded in 1967–1969 [6]. The most recent epidemic occurred in 1986/87 with 56 reported cases per 1,000 persons in malaria-endemic areas. During the 1990s, 70% of the reported cases were from Northern and Eastern Provinces of Sri Lanka [7]. The number of reported cases and deaths declined more than ten-fold since 1999 and in 2008, during which 196 confirmed cases of malaria were reported. In 2012, 24 indigenous and 70 imported malaria cases were reported and no case of indigenous malaria has been reported since October 2012 [8-10]. The end of the civil conflict in 2009 and implementation of intense malaria control activities in the Northern and Eastern Provinces and the neighbouring districts by the Anti Malaria Campaign (AMC), together with close monitoring and evaluation of interventions, may have contributed to this reduction and absence of cases.

Sri Lanka embarked on a malaria elimination programme in 2009 with the aim of eliminating *Plasmodium falciparum* by end 2012, and *Plasmodium vivax* by end 2014 [9]. Tropical and Environmental Disease and Health Associates Private Limited (TEDHA) is one of the three principal recipients of the Round 8 Global Fund to fight against AIDS, Tuberculosis and Malaria grant to assist the AMC in surveillance. TEDHA established malaria diagnostic laboratories in 43 government hospitals in the Northern and Eastern provinces which bore the brunt of the civil war, as requested by the AMC, to carry out parasitological surveillance to supplement the services carried out by the AMC. APCD, PCD and ACD have been used in Sri Lanka. APCD where all fever cases were tested for malaria was the mainstay of disease surveillance. With the reduction of the disease burden, doctors were reluctant to refer all fever cases for malaria testing and most of the time only suspected cases were referred for malaria testing making the system more PCD than APCD [11]. ACD has been used whenever outbreaks have occurred where mobile teams visited the areas and conducted surveys irrespective of whether persons had a history of fever or any symptoms suggestive of malaria.

All three surveillance techniques (PCD, APCD and ACD) are carried out by TEDHA by trained persons. This manuscript describes the importance of ACD in the malaria elimination efforts of Sri Lanka to interrupt malaria transmission.

## Methods

TEDHA screened populations using APCD, PCD and ACD. ACD was carried out through mobile malaria clinics (MMCs) including pregnant women visiting ante-natal clinics, and populations resident in close proximity to hospital surveillance sites by home visits.

A mobile malaria clinic (MMC) involves screening a pre-identified population for malaria parasites through active case detection. TEDHA conducted mobile malaria clinics using a trained team comprising a Parasitological Surveillance Officer (PSO) or a Fever Surveillance Officer (FSO), one or more Parasitological Surveillance Assistants (PSAs) and one or more Fever Surveillance Assistants (FSAs) depending on the number of people expected at a MMC, a MMC aide and a driver. FSOs and FSAs assist in the preparation of blood smears and the PSO’s and PSA’s stain and microscopically examine blood smears. A minimum of 50 persons were screened during a mobile clinic. MMCs were carried out among high risk groups. High risk groups were defined as 1) persons resident in traditionally malarious areas based on previous data where there is a high risk of malaria transmission, 2) population groups engaged in high risk behaviours such as the armed forces, chena cultivators involved in seasonal slash and burn cultivation, gem miners, etc. 3) displaced and migratory populations, 4) newly settled areas under the purview of development projects and re-settled populations following the end of the civil conflict terrorist war, 5) populations in which malaria cases have been detected, and 6) populations resident in inaccessible or remote areas with no access to malaria microscopy facilities.

Based on the above criteria, populations for MMCs were selected in consultation with the Regional Malaria Officer of the Anti-Malaria Campaign. Identified locations were visited by the Community Mobilisation Officer of TEDHA and the local head of the area, usually the Grama Niladhari Officer (GN officer), the government official in charge of the smallest administrative unit of the country, was apprised of the proposed activity. Having obtained the consent of the GN officer, the community was informed of the MMC through community leaders and arrangements were made to conduct the MMC at a suitable location, usually a school, a community centre, military units or a place of religious worship. On the assigned dates, clinics were commenced at 8:00 hrs by the MMC team.

In addition, TEDHA screened pregnant women visiting ante-natal clinics conducted in the areas for the first time in their current pregnancy for malaria. These women were not screened again during the pregnancy except if they were suspected of having malaria. TEDHA also conducted ACD in residents close to TEDHA assigned hospital sites by visiting households after giving prior notice of the visit. This type of surveillance are referred as ‘hospital village clinic’ (HVC) surveillance visits.

A finger prick blood sample was obtained from all individuals who participated in the ACD programme under sterile conditions using disposable equipment. Thick and thin blood smears were prepared, stained with Giemsa and examined under the microscope on site. One hundred fields of a thick smear were screened prior to reporting. If the thick smear was positive, 200 fields of a thin smear were screened for species identification. A report of the result was issued to all participants prior to the departure of the team. Whenever the number of blood smears taken could not be examined on site, they were taken to the TEDHA District Office and examined; reports of the blood smears were delivered to the participants the next day. Ten percent of randomly selected negative slides from each MMC and all positive slides were cross-checked by external quality control experts comprising senior Laboratory Technicians with over 10 years experience in malaria diagnosis from the Anti-Malaria Campaign and Departments of Parasitology of Medical Faculties.

Positive cases based on blood smear examination were referred to the closest government hospital for treatment. All vivax cases were treated with a course of chloroquine 25 mg/kg over a period of three days (10 mg/kg body weight on days 1 and 2, and 5 mg/kg body weight on day 3) and primaquine 0.25 mg/kg body weight for 14 days; all falciparum cases and mixed infections were treated in hospital with a fixed dose oral combination tablets of artemether (20 mg) and lumefantrine (120 mg) (Coartem^©^) based on body weight in six doses over a period of three days. For patients with a body weight of 35 kg, an initial dose of three tablets was administered, followed by three tablets after eight hours and three tablets twice daily (morning and evening) for the following two days (total course of 18 tablets). For persons <35 kg, an appropriate fixed dose was administered [12]. In addition to Coartem^©^ 0.75 mg/kg primaquine was given as a single dose on day 3 or prior to discharge from hospital [12]. All positive cases were notified to the Anti-Malaria Campaign and were followed up for 28 days as per WHO guidelines for clinical and parasitological response to treatment. As private pharmacies are reluctant to store antimalrial drugs as they move slowly and as government institutions are well stocked with antimalarials which are provided free of charge, it is unlikely that people will have access to antimalarials. As most of the population is literate and as most persons seek advice on health care especially as it is provided free of charge in government institutions, self-medication is very unlikely.

## Results

**Figure 1:**
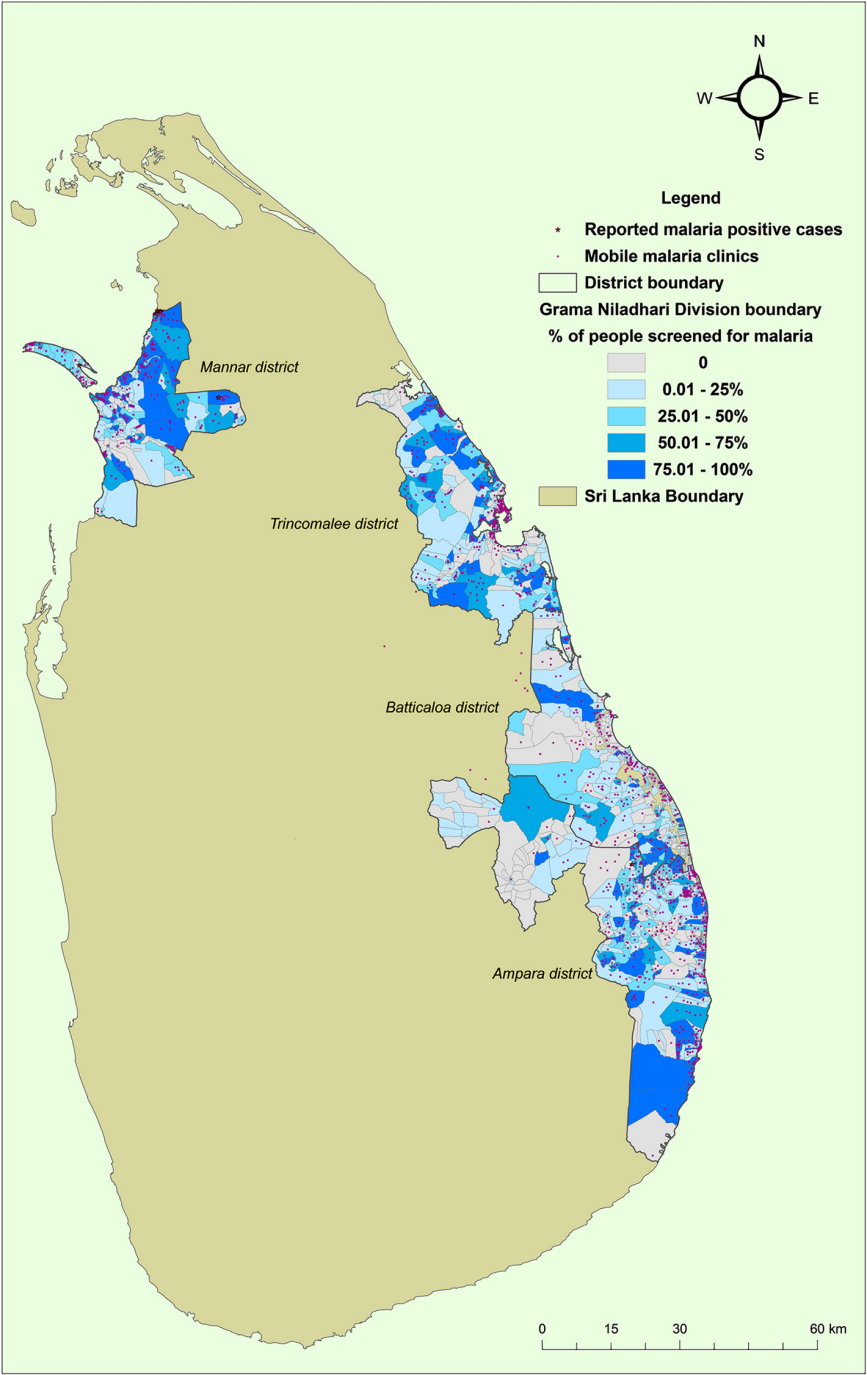
**The distribution of the population screened by active case detection and Grama Niladari Division.** The four districts where malaria surveillance was carried out are indicated on the map. The red stars indicate the positive cases and the red dots indicate the mobile malaria clinics.

TEDHA screened 387,309 individuals for malaria by ACD from January 2010 to December 2012 in the 4 districts. Table 1 gives the breakdown of the distribution of the population screened by ACD by age and sex. Figure 1 gives the distribution of the population screened by ACD and GN division.

**Table 1:** Population screened by active case detection (February 2010 to December 2012)

**District**	**Male N (%)**	**Female N (%)**	**Total N (%)**
Ampara			
<6 years	5,546 (52.5)	5,020 (47.5)	10,566 (100)
6-15 years	28,132 (51.9)	24,045 (48.1)	54,177 (100)
>15 years	38,876 (40.2)	57,719 (59.8)	96,595 (100)
Total	72,554 (45.0)	88,784 (55.0)	161,338 (100)
Batticaloa			
<6 years	1,810 (51.6)	1,700 (48.4)	3,510 (100)
6-15 years	13,612 (53.2)	11,991 (46.8)	25,603 (100)
>15 years	21,737 (50.2)	21,522 (49.8)	43,259 (100)
Total	37,159 (51.3)	35,213 (48.7)	72,372 (100)
Mannar			
<6 years	491 (48.9)	513 (51.5)	1,004 (100)
6-15 years	9,376 (50.5)	9,198 (49.5)	18,574 (100)
>15 years	14,492 (73.5)	5,220 (26.5)	19,712 (100)
Total	24,359 (62)	14,931 (38)	39,290 (100)
Trincomalee			
<6 years	3,798 (51.8)	3,532 (48.2)	7,330 (100)
6-15 years	23,514 (51.6)	22,018 (48.4)	45,532 (100)
>15 years	32,510 (53)	28,837 (47)	61,347 (100)
Total	59,822 (52.4)	54,387 (47.6)	114,209 (100)
All four districts			
<6 years	11,645 (52.0)	10,765 (48.0)	22,410 (100)
6-15 years	74,634 (51.9)	69,252 (48.1)	143,886 (100)
>15 years	107,615 (48.7)	113,298 (51.3)	220,913 (100)
Total	193,894 (50.1)	193,315 (49.9)	387,209 (100)

Since the commencement of surveillance operations in the four districts in February 2010, TEDHA detected nine malaria infections upto December 2012. Eight individuals were detected with *Plasmodium vivax* infections and one individual had a mixed infection of *P. vivax* and *P. falciparum*. Of the nine infections detected, eight were detected by ACD at MMCs and home visits conducted in the Mannar district without any symptoms (Figure 1). Of those detected by ACD, three were army personnel from the Vellankulum army camp; four were civilians from Sinnavalayankaddu, a re-settled village in the Mannar district and one was a civilian from Errukalampiddy (Table 2). Of the civilians from the re-settled village two were under the age of five years, of whom, one was a child of a positive mother of the same household. One civilian businessman from Errukalampiddy had returned from India five weeks prior to diagnosis, the rest did not report any travel overseas one month prior to diagnosis. Two patients had sought western medical treatment prior to diagnosis of malaria by TEDHA. The Health Care Providers had not requested for a blood smear for malaria. All military persons gave no history of travel abroad. Of those positive, only one civilian had gametocytes on blood smear examination; all the others had asexual stages of the parasites.

**Table 2:** Details of patients detected by active case detection from February 2010 to December 2012

**Case**	**Date**	**Location**	**Sex**	**Age**	**Diagnosis**
1	02.02.2010	Vellankulum army camp Mannar	Male	19	*Plasmodium vivax*
2	23.06.2010	Vellankulum army camp Mannar	Male	21	*Plasmodium vivax*
3	13.07.2010	Vellankulum army camp Mannar	Male	22	*Plasmodium vivax*
4	01.10.2010	Sinnavalayankaddu Mannar	Female	2	*Plasmodium vivax*
5	01.10.2010	Sinnavalayankaddu Mannar	Female	48	*Plasmodium vivax*
6	15.10.2010	Sinnavalayankaddu Mannar	Male	24	*Plasmodium vivax*
7	09.05.2011	Errukalampiddy Mannar	Male	37	*Mixed infection*
8	10.08.2011	Sinnavalayankaddu Mannar	Female	3	*Plasmodium vivax*

## Discussion

The importance of surveillance by Active Case Detection through TEDHA’s experience in implementing the Sri Lanka malaria elimination programme is demonstrated in this manuscript. Eight of the nine infections detected during the study period were through Mobile Malaria Clinics and home visits. The detection of malaria in Army personnel and among persons in a re-settled village implies the importance of carrying out surveillance in high risk populations. The criteria used for selecting sites for mobile malaria clinics were based on eliminating refractory foci of malaria transmission. No indigenous case of malaria has been reported in Sri Lanka since October 2012.

Although mobile malaria clinics were conducted in many parts of Mannar, a traditionally malaria endemic area, only one individual diagnosed with malaria reported travel overseas or outside the area. Since the reduction in the incidence of malaria cases in the country, the percentage of imported malaria cases has been rising. In 2013, there were 95 imported malaria cases reported with infections acquired mainly by travelers to India. All these cases were diagnosed by PCD with the majority being diagnosed after 5 days of the onset of symptoms. Positive infections that were found in this study were from two localities in the Mannar District, indicating that indigenous transmission had occurred. The source of infection is likely to be asymptomatic persons harbouring the parasites in low numbers, the detection of whom is a major challenge to an elimination drive.

Given the fact that detection of persons with low parasitaemias is a major challenge to a malaria elimination programme, targeted drug administration to other household members and close neighbours warrants consideration. In malaria elimination programmes, suspected cases are not considered in the nomenclature of infections and as such giving presumptive treatment in such instances is likely to be contradictory. It may be more appropriate to screen other household members and close neighbours with more sensitive diagnostic tests such as PCR and then treat if found positive. In Azerbaijan, Tajikistan (formerly USSR), North Afghanistan and DPR Korea, 8,270,185 people received either a 14-day “standard” or a 17-day “interrupted” primaquine treatment to control post-eradication malaria epidemics mainly targeting hypnozoites of *P. vivax*[13].

The Anti-Malaria Campaign in 2011 detected 21% of cases through ACD. In 2011, 124 cases of indigenous malaria and 51 cases of imported malaria were reported in the entire country [14]. Of the indigenous malaria cases, 22.1% of cases were detected through ACD by the AMC [14]. In the districts that TEDHA operated, the AMC conducted surveillance in large hospitals; TEDHA surveillance was confined to small rural hospitals. This probably explains why TEDHA detected 87.5% of cases by ACD as compared to 22.1% by the AMC as symptomatic patients are likely to visit large hospitals for consultation and treatment.

The fact that there was only one case of *P. falciparum* as a mixed infection in May 2011 implies that *P. falciparum* may have been on the verge of elimination; the national target for elimination of indigenous *P. falciparum* was the end of 2012. It is possible that *P. vivax* infections may have been relapses or fresh infections. All patients were treated as per guidelines of the AMC and followed over a period of 28 days [12]. All of the patients showed good clinical and parasitological response to treatment. As all patients, excluding one, did not have gametocytes in their peripheral blood, and as all infections were *P. vivax* infections, given the characteristics of *P. vivax* infections in which gametocytes appear early and respond efficiently to antimalarial drugs, it may be concluded that they were detected early resulting in a possible interruption of local transmission.

In recent years, malaria control has received much attention, including increased resources and partnerships such as from TEDHA which have helped in the implementation of malaria control interventions resulting in reductions in the disease burden in many parts of the world including Sri Lanka [1]. Grants received from the Global Fund to fight AIDS, Tuberculosis and Malaria (GFATM) over the years have augmented, supplemented and supported malaria control activities in Sri Lanka [9] and probably played a significant role in Sri Lanka embarking on a malaria elimination drive.

The progress made by Sri Lanka in the malaria elimination drive is largely due to increased surveillance and judicious use of control methods. The country now needs to focus on maintaining the momentum with enhanced surveillance for Sri Lanka to stay malaria free and to prevent its re-introduction into the country. For this to realize, as highlighted here, ACD will play a major role in the coming 3–4 years to ensure that reservoirs of parasites are detected and treated early, for which adequate funds and resources should be allocated as a priority.

## Conclusions

In this study, the importance of surveillance by Active Case Detection in implementing a malaria elimination programme is highlighted. The detection of malaria in Army personnel and among persons in a re-settled village implies the importance of carrying out ACD in high risk population groups.

## Abbreviations

ACD: Active case detection; AMC: Anti-malaria campaign; APCD: Activated passive case detection; GN: Grama Niladari; MMC: Mobile malaria clinics; PCD: Passive case detection; TEDHA: Tropical and Environmental Disease and Health Associates Private Limited.

## Competing interests

The authors declare that they have no competing interests.

## Authors’ contributions

RW was involved in drafting the manuscript, and in acquisition of data. SDF was involved in revising the manuscript and in acquisition of data. JT was involved in preparing the maps. PMW was involved in revising the manuscript. ARW was involved in the conception and the design of the manuscript, acquisition of data, analysis and interpretation of data and finalizing the manuscript. All authors contributed to writing the paper and read and approved the final manuscript.
